# The 24 h pattern of arterial pressure in mice is determined mainly by heart rate‐driven variation in cardiac output

**DOI:** 10.14814/phy2.12223

**Published:** 2014-11-26

**Authors:** Theodore W. Kurtz, Heidi L. Lujan, Stephen E. DiCarlo

**Affiliations:** 1 Department of Laboratory Medicine, University of California, San Francisco, San Francisco, California; 2 Department of Physiology, Wayne State University School of Medicine, Detroit, Michigan

**Keywords:** 24‐h variability, hemodynamics

## Abstract

Few studies have systematically investigated whether daily patterns of arterial blood pressure over 24 h are mediated by changes in cardiac output, peripheral resistance, or both. Understanding the hemodynamic mechanisms that determine the 24 h patterns of blood pressure may lead to a better understanding of how such patterns become disturbed in hypertension and influence risk for cardiovascular events. In conscious, unrestrained C57BL/6J mice, we investigated whether the 24 h pattern of arterial blood pressure is determined by variation in cardiac output, systemic vascular resistance, or both and also whether variations in cardiac output are mediated by variations in heart rate and or stroke volume. As expected, arterial pressure and locomotor activity were significantly (*P* < 0.05) higher during the nighttime period compared with the daytime period when mice are typically sleeping (+12.5 ± 1.0 mmHg, [13%] and +7.7 ± 1.3 activity counts, [254%], respectively). The higher arterial pressure during the nighttime period was mediated by higher cardiac output (+2.6 ± 0.3 mL/min, [26%], *P* < 0.05) in association with lower peripheral resistance (−1.5 ± 0.3 mmHg/mL/min, [−13%] *P* < 0.05). The increased cardiac output during the nighttime was mainly mediated by increased heart rate (+80.0 ± 16.5 beats/min, [18%] *P* < 0.05), as stroke volume increased minimally at night (+1.6 ± 0.5 *μ*L per beat, [6%] *P* < 0.05). These results indicate that in C57BL/6J mice, the 24 h pattern of blood pressure is hemodynamically mediated primarily by the 24 h pattern of cardiac output which is almost entirely determined by the 24 h pattern of heart rate. These findings suggest that the differences in blood pressure between nighttime and daytime are mainly driven by differences in heart rate which are strongly correlated with differences in locomotor activity.

## Abstract (precis)

In C57BL/6J mice, the 24 h pattern of blood pressure is hemodynamically mediated primarily by the 24 h pattern of cardiac output which is almost entirely determined by the 24 h pattern of heart rate. These findings suggest that the differences in blood pressure between nighttime and daytime are mainly driven by differences in heart rate which are strongly correlated with differences in locomotor activity.

## Introduction

The risk of occurrence of adverse cardiovascular events varies during the 24 h day. Incidents of myocardial ischemia, infarction, and cerebrovascular accidents occur more frequently during early morning hours than during any other times of the 24 h day (Bristow et al. 1969; Muller et al. 1985; Mulcahy et al. 1988; Marler et al. 1989; Mancia 1993; Veerman et al. 1995; Kario 2010). Similarly, there are variations in arterial blood pressure, heart rate, plasma norepinephrine levels, platelet aggregation, and fibrinolytic activity over the 24 h day (Muller et al. 1985; Andreotti et al. 1988; Panza et al. 1991). For example, arterial blood pressure and heart rate in humans are lower during the nighttime inactive period when sleep and rest predominate than during the daytime period when wakefulness and activity predominate. Furthermore, arterial blood pressure and heart rate increase sharply at the moment when humans arise in the morning (Millar‐Craig et al. 1978; Mancia et al. 1983; Leary et al. 2002; Kario 2010). It is possible that these variations in arterial blood pressure and heart rate contribute to the variation in risk for adverse cardiovascular events observed during the 24 h day.

The possibility that variations in arterial blood pressure and heart rate over 24 h may contribute to the variation in cardiovascular risk over 24 h has stimulated intense interest in factors that regulate 24 h rhythms of blood pressure and heart rate. Understanding the hemodynamic mechanisms that determine the 24 h patterns of blood pressure may lead to a better understanding of how such patterns become disturbed in hypertension and influence risk for cardiovascular events. Blood pressure is hemodynamically determined by cardiac output and total peripheral resistance; however, few studies in humans or in animals have systematically investigated whether daily patterns of arterial blood pressure over 24 h are mediated by changes in cardiac output, peripheral resistance, or both. In the mouse, a species that is widely used to investigate genetic and behavioral determinants of various biologic rhythms including blood pressure (Mitler et al. 1977; Van Vliet et al. 2003, 2006; Silvani et al. 2009, 2010; Wang et al. 2010; Bastianini et al. 2011, 2012), the daily patterns of cardiac output and peripheral resistance over 24 h have not been reported. Thus, in the mouse, it is unknown whether the 24 h patterns of arterial blood pressure are mediated by variation in cardiac output, peripheral resistance, or both. Therefore, in the current study in C57BL/6J mice, we investigated the hemodynamic determinants of the 24 h pattern of arterial blood pressure by continuously recording arterial pressure, cardiac output, heart rate, peripheral resistance, and stroke volume over a period of 5 days.

## Materials and Methods

### Animals and general experimental procedures

Experimental procedures and protocols were reviewed and approved by the Animal Care and Use Committee of Wayne State University and complied with The American Physiological Society's Guiding Principles in the Care and Use of Animals. The procedures were conducted in 10 male C57BL/6J mice (3–4 months of age). Mice were instrumented with an intra‐arterial telemetry device (PA‐C10; Data Sciences International, St. Paul, MN) for recording arterial pressure, heart rate, and locomotor activity and with an ascending aorta Doppler ultrasonic flow probe for recording cardiac output. After recovery, continuous 24‐h patterns of mean arterial pressure, cardiac output, heart rate, peripheral resistance, stroke volume, and locomotor activity were obtained over 5 consecutive days and nights as described below.

### Surgical procedures

All surgical procedures were performed using aseptic surgical techniques. Mice were anesthetized with pentobarbital sodium (50 mg/kg ip), atropinized (0.05 mg/kg ip), intubated, and prepared for aseptic surgery. Supplemental doses of pentobarbital sodium (10–20 mg/kg ip) were administered if the mice regained the blink reflex or responded during the surgical procedures.

The hearts were approached via a left thoracotomy through the second intercostal space (Lujan et al. 2012a,b; Lujan and DiCarlo 2013, 2014a,b). Subsequently, the sleeve of the pericardium that extends onto the ascending aorta was dissected free of surrounding tissue and a 1.6‐mm silicone‐type Doppler ultrasonic flow probe (Iowa Doppler Products, Iowa City, IA) was positioned around the ascending aorta (Lujan and DiCarlo 2013, 2014a,b). The flow probe wires were tunneled subcutaneously and exteriorized at the back of the neck. Subsequently, a catheter from a telemetry device (PA‐C10; Data Sciences International), for recording arterial pressure and locomotor activity, was inserted into the left carotid artery until the tip reached the aortic arch.

All animals remained on the feedback‐based temperature control system and ventilator until recovered from the anesthesia. Once the animals regained consciousness, they were placed in a “rodent recovery cage” (Thermocare^®^ Intensive Care Unit; Braintree Scientific, Braintree, MA). Animals were returned to the housing room when fully recovered from the anesthesia and gained the ability to maintain body temperature. The nonsteroidal anti‐inflammatory agent ketoprofen (5 mg/kg, sq), and the antibiotic cefazolin (10 mg/kg, sq) were continued for 2 days. At least 10 days were allowed for recovery. During the recovery period, the mice were handled, weighed, and acclimatized to the laboratory and investigators.

### Determination of study variables: arterial pressure, cardiac output, heart rate, peripheral resistance, stroke volume, and locomotor activity

Mice were housed under a 12 h:12 h Light/Dark cycle with ambient temperature monitored and maintained near the thermoneutral zone for mice. Mice are very responsive to variations in ambient temperature and are often studied in rooms maintained between 21 and 23°C, which is markedly below their thermoneutral zone of 30°C (Swoap et al. 2004). Studies conducted in rooms with temperatures below thermoneutrality serve as a mild cold stress, and the mice respond with tachycardia due to a withdrawal of cardiac vagal tone and increased sympathetic tone (Swoap et al. 2004). Thus, in the current study, examining responses in an environment within the thermoneutral zone for mice likely reflects the basal hemodynamic state (Swoap et al. 2004).

**Figure 1.**
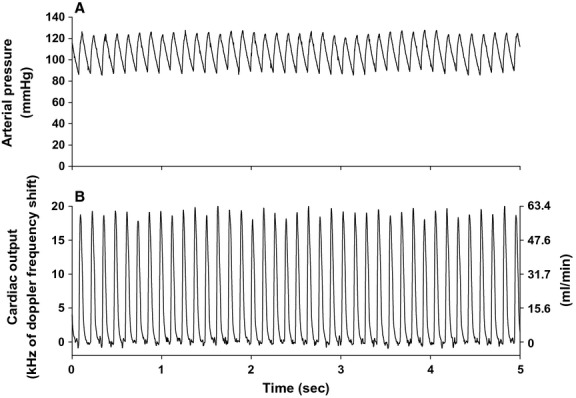
High‐fidelity 5‐sec original recordings of arterial pressure (panel A) and ascending aortic blood flow (cardiac output, panel B) during the daytime resting period from a chronically instrumented mouse are shown. Units for cardiac output (panel B) are expressed in kHz (left *y*‐axis) and mL/min (right *y*‐axis).

Cardiac output was recorded by securing the flow probe leads to multi‐stranded stainless steel wires from a miniature electric swivel (catalog # FL‐2‐C‐Micro; Dragonfly Research and Development, INC., Ridgeley, WV) and the mouse cage was placed on radio‐telemetry receivers. This permitted 24‐h recording of arterial pressure, heart rate, cardiac output, and locomotor activity (Fig. 1). Total peripheral resistance and stroke volume were calculated as described below.

### Data recording and analysis

**Figure 2.**
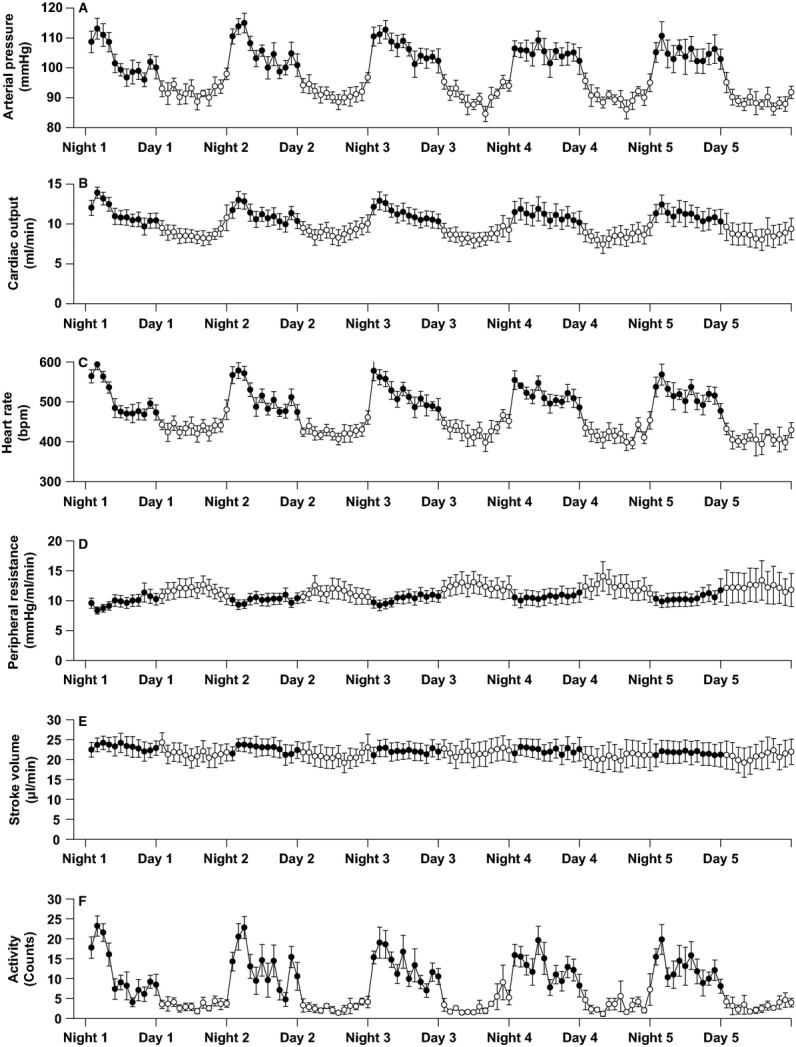
Daytime (6 am–6 pm) and nighttime (6 pm–6 am) hourly averages in arterial blood pressure (panel A), cardiac output (panel B), heart rate (panel C), peripheral resistance (panel D), stroke volume (panel E), and locomotor activity (panel F) determined over 5 consecutive 24 h periods. Open circles denote the hourly averages during the daytime period in which rest and sleep predominate and solid circles denote the hourly averages during the nighttime period when wakefulness and activity predominate. Data are presented as means ± SEM.

**Figure 3.**
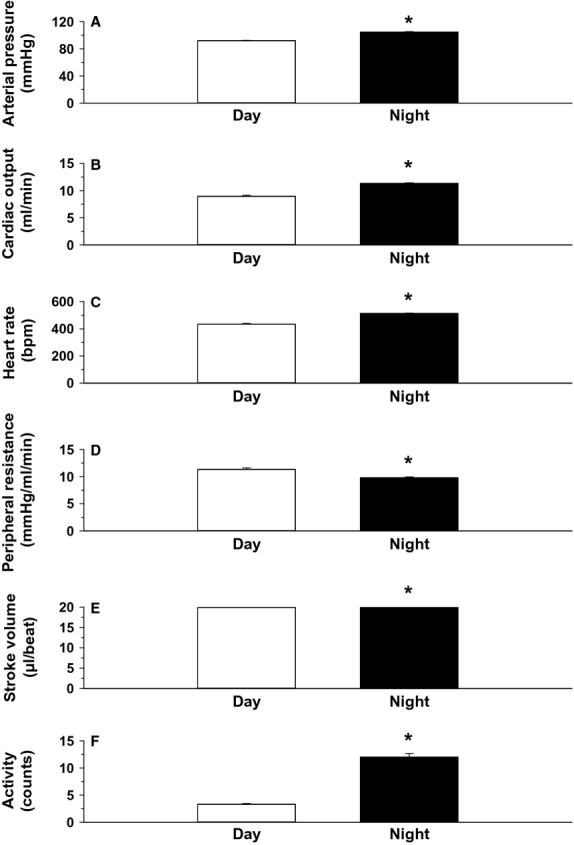
The 12 h daytime and 12 h nighttime mean values for each variable determined over the 5 consecutive 24 h periods. Data are presented as means ± SEM. *P < 0.05, night versus day. The absolute and percent differences between the paired daytime and nighttime values are shown in Table 1.

All physiological recordings were sampled continuously at 2 kHz, beat to beat 24 h/day for 5 consecutive days and nights. Subsequently, 1 h averages for each variable were calculated over each 24 h period and are presented in Figure 2. In addition, the 12 h daytime and nighttime averages were obtained for each animal for each of the five 24 h sampling periods. The 12 h averages from these five 24 h periods were averaged for each animal to yield the study average daytime and nighttime values for each animal. The study average values from the individual animals were also averaged to give the group mean study values for the daytime period and for the nighttime period (Fig. 3). Paired *t*‐tests were used to compare the study average daytime versus nighttime values within the animals. To obtain nighttime–daytime differences for each variable, the difference between the 12 h nighttime average minus the 12 h daytime average was calculated for each animal for each of the five 24 h periods. These nighttime–daytime differences in each of the five 24 h periods were then averaged to obtain the study average nighttime–daytime difference for each animal. These study average nighttime–daytime differences from each animal were then averaged to give the group means for the nighttime–daytime differences. Data are expressed as means ± SE.

Pearson product moment correlations were used to measure the degree of linear dependence between the average levels of locomotor activity each hour versus the average levels of heart rate, cardiac output, arterial pressure, and peripheral resistance each hour. A *P* value of <0.05 was considered statistically significant.

### Calculations

Cardiac output was evaluated using an ultrasonic range‐gated pulsed Doppler flow meter (Hartley and Cole 1974). Blood flow (*Q*), in mL/min, was calculated by:
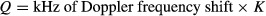
where *K *=**1.24 × *d*^2^, (where *d* is the cuff diameter of the flow probe measured in millimeters). Using this system, the Doppler shift frequency (kHz) is directly proportional to blood flow (Hartley and Cole 1974).

Total peripheral resistance was calculated by:
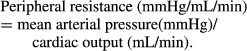


Stroke volume (SV), in *μ*L/beat, was calculated by:
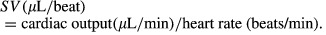


## Results

Original recordings of arterial pressure and ascending aortic blood flow (cardiac output) in an intact, conscious, mouse are shown in Figure 1. High‐fidelity recordings of this nature were obtained in all animals throughout the study. The hourly averages for arterial blood pressure (panel A), cardiac output (panel B), heart rate (panel C), peripheral resistance (panel D), stroke volume (panel E), and locomotor activity (panel F) over 5 consecutive days and nights are presented in Figure 2. The 12 h daytime and 12 h nighttime mean values for each variable are shown in Figure 3. For each variable, significant differences were observed between the 12 h daytime value versus the 12 h nighttime value (Fig. 3). Specifically, arterial pressure, heart rate, cardiac output, stroke volume, and locomotor activity were significantly greater in the 12 h nighttime period than in the 12 h daytime period (Fig. 3). Peripheral resistance was significantly lower in the 12 h nighttime period than in the 12 h daytime period (Fig. 3). Table 1 shows the mean absolute differences and percentage differences between the paired nighttime values – the daytime values for each variable. The results demonstrate that the increase in arterial pressure (~13%) during the nighttime active period is mediated by an increase in cardiac output (~26%) in association with a decrease in peripheral resistance (~−13%). The increased cardiac output during the nighttime was mainly mediated by increased heart rate (~18%) as stroke volume increased only slightly at night (~6%). Significant (*P* < 0.05) positive correlations were observed between the average levels of locomotor activity each hour and the average levels of heart rate (*r*^2^ = 0.944), cardiac output (*r*^2^ = 0.941), and arterial pressure (*r*^2^ = 0.936) each hour. A significant (*P* < 0.05) negative correlation was observed between locomotor activity and peripheral resistance (*r*^2^ = −0.822).

**Table 1.** The absolute and percent differences between the paired daytime and nighttime values.

Variable	Δ Night–Day (absolute)	Δ Night–Day (percent)
Arterial pressure (mmHg)	12.5 ± 1.0	+13
Peripheral resistance (mmHg/mL/min)	−1.5 ± 0.3	−13
Cardiac output (mL/min)	2.6 ± 0.3	+26
Heart rate (beats/min)	80.0 ± 16.5	+18
Stroke volume (*μ*L/beat)	1.6 ± 0.5	+6
Locomotor activity (counts)	7.7 ± 1.3	+254

Δ Night–Day (absolute): Mean of the differences in the 12 h nighttime values (active period) – 12 h daytime values (mean ± SEM).

## Discussion

In humans, arterial blood pressure is lower during the nighttime period when sleep and rest predominate than during the daytime period when wakefulness and activity predominate. The factors that determine the variation in blood pressure over the 24 h period are of considerable interest because the patterns of blood pressure over 24 h may influence cardiovascular risk and may also be relevant to pharmacologic approaches to the management and prevention of target organ damage (Mancia and Parati 2006; Yano and Kario 2012; Hermida et al. 2014a). For example, in humans, the surge in blood pressure that occurs during the morning is associated with an increased risk for adverse cardiovascular events (Atkinson et al. 2014). In addition, cardiovascular risk appears to be increased in individuals termed “non‐dippers” in whom blood pressure fails to undergo the normally expected reduction during the nighttime period of rest and sleep (O'Brien et al. 1988). Recent studies have indicated that administration of antihypertensive medication at bedtime may be more effective in protecting against cardiovascular events than administration of antihypertensive medication in the daytime (Hermida et al. 2014b). Nondipping of heart rate during the nighttime period of rest and sleep is also associated with increased cardiovascular risk (Eguchi et al. 2009).

Little information is available on the basic hemodynamic mechanisms that mediate the 24 h patterns of blood pressure in states of health and disease. Specifically, there is a paucity of studies examining the relationships between the 24 h pattern of blood pressure and the 24 h patterns of cardiac output and peripheral resistance in ambulatory humans or animals with or without hypertension. Moreover, there do not appear to be any previous reports showing continuous changes in cardiac output and peripheral resistance occurring throughout two or more consecutive 24 h periods. Because the mouse can be genetically manipulated and studied in ways that are not possible in humans, mice are frequently used to investigate the genetic and behavioral factors that influence the changes in blood pressure that occur between the daytime and nighttime periods (Mitler et al. 1977; Van Vliet et al. 2003, 2006; Silvani et al. 2009, 2010; Wang et al. 2010; Bastianini et al. 2011, 2012). In mice as in humans, arterial blood pressure is lower during the period when sleep and rest predominate (which is daytime for mice), than during the period when wakefulness and activity predominate (which is nighttime for mice; Fig. 2). However, the hemodynamic determinants of the 24 h blood pressure pattern in mice have not been reported and therefore, the relationships between the 24 h pattern of blood pressure and the 24 h patterns of cardiac output and peripheral resistance are unknown. Accordingly, it is also unknown whether the hemodynamic factors that mediate the 24 h blood pressure pattern in mice are the same or different from those that determine the 24 h patterns of blood pressure in humans or in other species.

In the current study, we used a radio‐telemetry method for continuously measuring blood pressure together with a miniaturized Doppler flow probe technique for continuously measuring cardiac output to determine the 24 h patterns of blood pressure, cardiac output, heart rate, stroke volume, and peripheral resistance in conscious, unrestrained mice over a period of 5 days. In C57BL/6J mice, we found that the increase in blood pressure that occurs during the period of wakefulness and activity is hemodynamically determined by an increase in cardiac output in association with a decrease in peripheral resistance. The increase in cardiac output is mainly determined by an increase in heart rate which is strongly correlated with increased locomotor activity. Thus, in C57BL/6J mice, the blood pressure pattern over 24 h is largely determined by changes in cardiac output that are mainly driven by changes in heart rate associated with changes in locomotor activity.

In humans, the hemodynamic determinants of the pattern in arterial pressure over 24 h are inconclusive (Khatri and Freis 1967; Bristow et al. 1969; Miller and Horvath 1976; de Zambotti et al. 2012) possibly due to the fact that only one study determined patterns in arterial pressure, cardiac output, and vascular resistance in ambulatory subjects for an entire 24 h period (Veerman et al. 1995). However, in that one study in humans, as in our study in mice, the blood pressure pattern over 24 h was also mainly determined by changes in cardiac output that were driven by changes in heart rate. The increase in arterial pressure during the period of wakefulness and activity (i.e., daytime in humans) was hemodynamically determined by an increase in cardiac output in association with a decrease in peripheral resistance; the increase in cardiac output was attributed to an increase in heart rate, not in stroke volume (Veerman et al. 1995).

In unanesthetized, unrestrained rats studied for a full 24 h, and in few monkeys studied for periods of 18 h while chronically restrained in chairs, the increases in blood pressure associated with the periods of wakefulness and activity have also been associated with increases in cardiac output, reductions in peripheral resistance, and increases in heart rate with little or no change in stroke volume (Engel and Talan 1987; Smith et al. 1987). In dogs, increases in blood pressure during the morning are reported to be due to increases in cardiac output in association with little or no change in peripheral resistance (Anderson et al. 1990). In contrast to the results in mice, rats, humans, and monkeys, the increases in cardiac output in dogs that occur when wakefulness predominates appear to be due to increases in stroke volume, not increases in heart rate (Anderson et al. 1990).

In the current study, sharp increases in cardiac output occurred within the first hour of the transition from the inactive period to the active period. These sharp increases in cardiac output were largely driven by increases in heart rate and were mainly responsible for the sharp increases in blood pressure that occurred during this transition phase. These observations are consistent with the idea that changes in arousal and locomotor activity are largely responsible for the hemodynamic changes that occur between the period in which rest and sleep predominate and the period when wakefulness and activity predominate (Mann et al. 1979; Rowlands et al. 1980; Clark et al. 1987; Veerman et al. 1995; Leary et al. 2002; Van Vliet et al. 2003, 2006). The onset or offset of sleep itself appears to have relatively little effect on blood pressure compared to the blood pressure effects of changes in mental and locomotor activity that take place before the onset of sleep, or after waking (Leary et al. 2002; Carrington et al. 2003; Zaregarizi et al. 2007). Although endogenous circadian rhythms influencing blood pressure and other hemodynamic patterns have been reported in mice as in humans, the overall contribution of an endogenous rhythm to the 24 h blood pressure pattern appears modest and the pattern seems to be largely determined by changes in levels of arousal and activity (Curtis et al. 2007; Zuber et al. 2009; Sheward et al. 2010; Vukolic et al. 2010; Wang et al. 2010; Shea et al. 2011).

It would seem reasonable to speculate that during transition from the inactive period to the active period, increases in arousal may lead to neurally mediated increases in heart rate that determine the increases in cardiac output we observed to mediate increases in blood pressure. Increased demand for oxygen stimulated by increases in activity may be a further determinant of the increased cardiac output and decreased peripheral resistance that together determined the magnitude of the increase in blood pressure during transition from the inactive period to the active period. It is expected that the technology we describe for continuously monitoring blood pressure, cardiac output, heart rate, stroke volume, and peripheral resistance over extended periods will enable future studies to identify the hemodynamic mechanisms that mediate disturbances in the 24 h blood pressure patterns known to be associated with increased cardiovascular risk in disorders such as hypertension.

## Perspective

Although 24 h patterns of blood pressure have been extensively studied in animals and humans including in those with cardiovascular disorders such as hypertension, little is known about the 24 h patterns of cardiac output and peripheral resistance that together determine the 24 h patterns of blood pressure in health or in disease. Investigation of the hemodynamic mechanisms that determine the 24 h patterns of blood pressure may lead to a better understanding of how such patterns become disturbed in hypertension and influence risk for cardiovascular events. This paper describes the use of Doppler flow probe technology and radio‐telemetry blood pressure monitoring techniques to determine the 24 h patterns of cardiac output and peripheral resistance which mediate the 24 h pattern of blood pressure in unanesthetized, unrestrained C57BL/6J mice. The current findings should open the door to future studies to determine the hemodynamic mechanisms that mediate disturbances of the 24 h patterns of blood pressure in hypertension and other disorders associated with increased risk of adverse cardiovascular events such as stroke and myocardial infarction. Given the large numbers of genetically manipulated strains of mice that are available, the techniques described in this manuscript should also be useful for determining the hemodynamic mechanisms whereby targeted genetic alterations influence the 24 h pattern of blood pressure. In addition, as proposed by Snelder and colleagues (Snelder et al. 2013, 2014), we anticipate that these kinds of studies of the 24 h patterns of cardiac output and peripheral resistance will be useful for investigating the hemodynamic mechanisms that mediate the effects of different antihypertensive drugs on 24 h blood pressure profiles. Finally, such studies may help determine if the manner in which an antihypertensive drug affects 24 h profiles of cardiac output and peripheral resistance can influence cardiovascular outcomes independent of drug effects on the 24 h blood pressure profile.

## Acknowledgment

We thank H. Janbaih for his expert technical assistance.

## Conflicts of Interest

No conflicts of interest, financial or otherwise, are declared by the authors**.**
